# A Historical Perspective of Bladderworts (*Utricularia*): Traps, Carnivory and Body Architecture

**DOI:** 10.3390/plants10122656

**Published:** 2021-12-03

**Authors:** Vitor F. O. Miranda, Saura R. Silva, Markus S. Reut, Hugo Dolsan, Piotr Stolarczyk, Rolf Rutishauser, Bartosz J. Płachno

**Affiliations:** 1 Laboratory of Plant Systematics, Department of Applied Biology, School of Agricultural and Veterinarian Sciences, Campus Jaboticabal, UNESP—São Paulo State University, Jaboticabal CEP 14884-900, Brazil; saura.silva@unesp.br (S.R.S.); hugo.dolsan@unesp.com (H.D.); 2 Department of Plant Cytology and Embryology, Institute of Botany, Faculty of Biology, Jagiellonian University in Kraków, Gronostajowa 9 St., 30-387 Kraków, Poland; info.reut@gmail.com (M.S.R.); bartosz.plachno@uj.edu.pl (B.J.P.); 3 Department of Botany, Physiology and Plant Protection, Faculty of Biotechnology and Horticulture, University of Agriculture in Kraków, al. 29 Listopada 54, 31-425 Kraków, Poland; stolarczykp@interia.pl; 4 Department of Systematic and Evolutionary Botany, University of Zurich, CH-8008 Zurich, Switzerland; rolf.rutishauser@systbot.uzh.ch

**Keywords:** carnivorous plants, Lentibulariaceae, *Utricularia*, bauplan, trap function, carnivory

## Abstract

The genus *Utricularia* includes around 250 species of carnivorous plants, commonly known as bladderworts. The generic name *Utricularia* was coined by Carolus Linnaeus in reference to the carnivorous organs (*Utriculus* in Latin) present in all species of the genus. Since the formal proposition by Linnaeus, many species of *Utricularia* were described, but only scarce information about the biology for most species is known. All *Utricularia* species are herbs with vegetative organs that do not follow traditional models of morphological classification. Since the formal description of *Utricularia* in the 18th century, the trap function has intrigued naturalists. Historically, the traps were regarded as floating organs, a common hypothesis that was maintained by different botanists. However, Charles Darwin was most likely the first naturalist to refute this idea, since even with the removal of all traps, the plants continued to float. More recently, due mainly to methodological advances, detailed studies on the trap function and mechanisms could be investigated. This review shows a historical perspective on *Utricularia* studies which focuses on the traps and body organization.

## 1. Introduction

*Utricularia* species (bladderworts) are one of the most fascinating carnivorous plants, with tiny traps and seemingly no roots [1,2,3,4,5,6,7,8]. The traps, also known as utricles, are foliar structures shaped in small vesicles, which are active in prey capture and the secretion of hydrolytic enzymes for digesting small prey, usually arthropods [9,10,11,12,13,14]. Intriguingly, the vegetative structures of all *Utricularia* species usually do not follow traditional models of morphological classification. The body organization of *Utricularia* does not present clearly defined boundaries between ordinary plant organs (stems and leaves, for instance), or recognizable roots [2], which make the organs’ recognition and classification a challenging work. In this sense, further studies about ontogeny and molecular genetics may provide information and tools for new perspectives in plant dynamics (cf. [8]). This mini review presents an overview of the bladderworts and aspects regarding their controversial body architecture and function from a historical perspective.

## 2. A Historical Reflection on the Bladderworts—From the First Description to the Present Day

**Figure 1:**
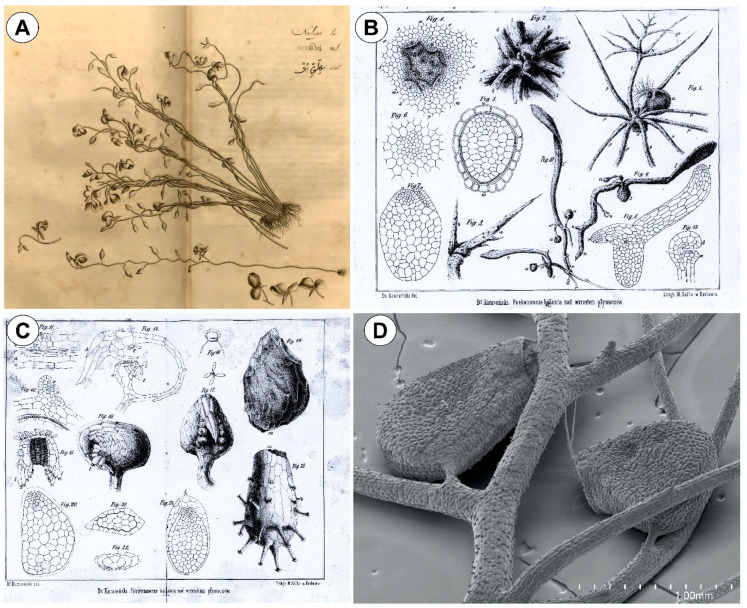
(**A**) One of the earliest records of *Utricularia* made by Hendrich van Rheede tot Draakenstein (1636–1691) in his *Hortus Malabaricus* (1689). According to Taylor [20], this is *Utricularia reticulata* Sm., a species with twining inflorescences, a “common weed of rice cultivation”. (**B**,**C**) Scanned figures from Franciszek Kamieński’s study which described in detail seed structure, seedlings, and traps of *Utricularia* species [34]. (**D**) The aquatic *Utricularia aurea* [35] with two traps by SEM (scanning electron microscopy–image by Bartosz J. Płachno).

The earliest illustrations of *Utricularia* were most likely by Conrad Gessner (1516–1565), a Swiss physician and naturalist, in his *Historia Plantarum*, also called *Conradi Gesneri Historia Plantarum* [15]. In *Libri Picturati,* (a collection of watercolors from the second half of the sixteenth century), there is one of *Utricularia* which is accessible at the Jagiellonian Library of the Jagiellonian University in Kraków [16]. The species shown in that watercolor is *Utricularia vulgaris* [10], in which the artist depicted flowers and fruits. However, traps on the vegetative parts were not shown. In his *Hortus Malabaricus* in 1689 [17], Hendrich Adriaan van Rheede tot Draakenstein (1636–1691), the Governor of the Dutch possession in Malabar (currently Kerala State), compiled a comprehensive work on the flora of the Western Ghats, which involved several naturalists and collaborators. In that treatise, he referred to *Utricularia reticulata* as “Nelipu” (Figure 1A), a Malayalam name that alludes to paddy fields where the species was found (Nellu = paddy; Pu = flower) [18]. “Nelipu” was probably the first non-European *Utricularia* to be referenced before Carolus Linnaeus (1707–1778), the founder of modern taxonomy, who proposed the genus *Utricularia* and described seven species in his *Species Plantarum* [19]: *U. vulgaris*, *U. minor*, *U. subulata*, *U. gibba*, *U. bifida*, *U. caerulea*, and *U. foliosa.* The *U. caerulea* treated by Linnaeus was a blend of different taxa [18,20], representing a specimen collected by Hermann in Ceylon, the plate presented by van Rheede in 1689 (Figure 1A), and a specimen named *U. caerulea* by Linnaeus himself [20].

The generic name *Utricularia* was coined by Linnaeus in reference to the traps (*utriculus* in Latin, meaning a womb or stomach) which are the carnivorous structures present in all species of the genus from aquatic to terrestrial environments [21,22]. The traps, historically known as utriculae (utricules), urceoli, ampullae, or bladders, are foliar structures shaped as small vesicles, which are active in prey capture and the secretion of hydrolytic enzymes adapted for digesting small animals [23]. Since the initial description of *Utricularia* by Linnaeus, many species have been described. In particular, Vahl [24], Brown [25], Smith [26], Saint Hilaire [27,28,29,30], and De Candolle [31] contributed to the addition of new species, mainly as a result of expeditions to the New World [32]. Several attempts have been made to split new genera from *Utricularia*, mostly by Rafinesque [33], who based his approach on inaccurate descriptions. Consequently, these new genera were not recognized and were thus synonymized under *Utricularia* by other authors [20].

In 1989, Peter Geoffrey Taylor (1926–2011), a British botanist who worked at the Royal Botanic Gardens, Kew, published his magnificent monograph about the genus *Utricularia* after dedicating four decades of his life to the bladderworts. Taylor’s [20] revision synonymized many taxa, and the number of species was reduced to 214. In recognition of his work on the genus, some taxa were named in honor of him, including two *Utricularia* species: the Indian *U. tayloriana* [36], synonymized by himself in his monograph (as *U. hirta*), and the Australian *U. petertaylorii* [37]. To date, the number of species has increased to around 250, mainly due to new discoveries in tropical America and Australia [38].

## 3. The “Nonconforming” and Dynamic Bauplan of *Utricularia*

The structural design (bauplan) of Lentibulariaceae and *Utricularia* has been highlighted as unique in many studies (e.g., [20,34,39,40,41,42,43]). Brugger and Rutishauser [44], Rutishauser and Sattler [45], Sattler and Rutishauser [46], and recently Reut and Płachno [8] presented meticulous studies of the organography and organogenesis of several *Utricularia* species.

All *Utricularia* species have a similar bauplan that lacks morphologically typical roots [47]. Some species exhibit branched shoots (stolons, or water shoots in aquatic species) that give rise to other (branched) shoots or stems, inflorescences, and traps (discussed in the next section). The floral architecture is usually similar to other snapdragon-like genera. The peduncle of the inflorescences arises from the shoots, and the gamopetalous flowers are zygomorphic. The androecium possesses two stamens, and the gynoecium consists of a superior ovary and one bilabiate stigma (e.g., [20,48]). Many terrestrial (e.g., *U. nervosa, U. subulata*), lithophytic (e.g., *U. flaccida*), rheophytic (e.g., *U. mirabilis*, *U. neottioides*), and some aquatic species (e.g., *U. intermedia*, *U. volubilis*) have “rhizoids” [20], which can be characterized as rather short and stiff shoots, with some having claw-like leaves [41,49]. In several aquatic species (e.g., *U. aurea*, *U. breviscapa*, *U. inflata*), a whorl of floats is found at the inflorescence base, resembling swollen shoots filled with aerenchyma cells and lacunae, and with appendages similar to branched shoots. These structures provide stability to the inflorescence, keeping it above the water table and making it more conspicuous to pollinators [20,35,50].

Contrary to the classical bauplan concept of vascular plants with the basic organ classes of shoot (stem and leaf) and root (e.g., [51]), the vegetative body of *Utricularia* shows organs with intermixed morphological traits (or developmental programs) of organ categories (e.g., [7,8]). For instance, unlike Goebel [39,40], Troll and Dietz [52] interpreted the “phyllomorphic organs” (“leaves”) of terrestrial *Utricularia* species as flattened shoots, i.e., phylloclades (as found in cacti), and homologous to shoots. This was especially evident to the authors in the dimorphic leaves of *U. dusenii* (syn. *U. nephrophylla sensu* Taylor [20]), of which the long and narrow type show a prolonged apical growth, which would generally be expected to occur in shoots such as stolons. Furthermore, Troll and Dietz [52] postulated that the stolons, due to their axillary nature (i.e., *quasi* in “invers axillary position”, cf. [8]), would combine leaf traits with shoot characters and therefore would also count as phylloclades. Combinations of characters of two extreme categories (leaf and stem) lead to intermediate organs with partial homologies, as postulated by Sattler and his school [53,54]. Similarly, although no organ of *Utricularia* morphologically shows typical root characters (such as a root cap, epidermal root unicellular hairs, or endogenous branching), few anatomical characters typical for roots are present in stolons ([2,7,8,44], Reut et al., in prep.). Therefore, unlike classic morphology, the vegetative body of *Utricularia* does not fit into narrowly defined positional and anatomical constraints and can be understood as morphologically “fuzzy”, following the Fuzzy Arberian Morphology approach, published by Agnes Arber between 1920 and 1957 [2]. Agnes Robertson Arber (1879–1960), a great admirer of Goethe’s drawings [55], was surely a leading figure of the era and anticipated several explanatory models for vascular plant development.

## 4. *Utricularia* Traps: Architecture and Function

The bladderwort’s trap is one of the most complex and intriguing structures among living plants (Figure 1D) and is far from being fully understood. Since the formal description of *Utricularia* in the 18th century, the trap structure and function has fascinated naturalists. Although Charles Darwin (1809–1882) is best known for his *The Origin of Species* [56], he passionately dedicated years of extensive experiments on carnivorous plants. In a letter [57] to the Scottish geologist Sir Charles Lyell in 1860, one year after the publication of *The Origin of the Species*, Darwin wrote: “I care more about *Drosera* than the origin of all the species in the world”. That sentence became more understandable when, after fifteen years, he published his *Insectivorous Plants* [58], which was a crucial reference work on carnivorous plants in the 19th century, and still is an important treatise in which Darwin meticulously described the structure and function of *Utricularia* traps.

Although the traps of *Utricularia* are similar in general organization and structure, they arise from different parts of the plant in different species: from the petiole and lamina of the leaves, from the base of inflorescences, and/or from stolons and their branches. The traps are foliar structures shaped as globose or ovoid vesicles (bladders) with a length of 0.2 to 12 mm [20,21,59,60]. Traps are usually translucent but are also pigmented in some taxa in greenish or reddish hues, due to the presence of chlorophyll or anthocyanin, respectively. In some species (e.g., *U. breviscapa*, *U. foliosa*, and *U. gibba*), the mature traps are even dark violet, most likely due to the presence of bacteria [61,62] or anthocyanins in vacuoles of trap wall cells.

Trap development has been the subject of several studies that attempted to anatomically and genetically understand the processes that result in such a complex structure (e.g., [7,21,63,64]). Lee et al. [65] proposed a robust 3D model for how leaf or leaflet primordia could generate the complex traps in *Utricularia* and hypothesized the presence of a polarity from stalk to mouth by analyzing the trap glands. Impressively, by controlling the adaxial–abaxial domains of gene activity for growth orientation in proximodistal and orthoplanar polarity in the primordia of *U. gibba*, simple shifts in the expression of genes homologous to the adaxial expressed PHV/PHB genes and abaxial expressed FIL and KAN genes [66,67,68] can induce the organ primordia to become filiform leaflets by tapering a cylindrical shape that becomes more wide than it is thick, or to become a trap by curving both longitudinally and transversally from a three-cell-thick layer. This becomes almost spherical with a closed mouth and results in a curved sheet, with adaxial domains on the inner regions and abaxial domains on the outer regions of the trap [69].

Historically, various botanists initially thought that the traps were flotation organs [26]. Nonetheless, Darwin [58] was one of the first naturalists to refute this idea, since even after the removal of all traps, the plants continued to float. It was only during the 19th century that some authors suggested that the traps had the ability to capture small animals, possibly as a nutrient source [70,71]. In [72], Holland wrote: “water insects often found imprisoned in the bladders destined for the plant to feed on” (in [73]). Nevertheless, Cohn [74] was the first botanist to prove the predatory ability of *Utricularia* by direct laboratory experimentation. It is now well established that traps actively capture prey and secrete hydrolytic enzymes suitable for digesting small animals [6,9,10,11,13,14,23].

All terrestrial, epiphytic, lithophytic, rheophytic, or aquatic *Utricularia* species have traps. Designating the rheophytic *U. neottioides* as “trapless” or “almost trapless” [75] is an incorrect assertion, since the fragile traps of this species are usually stuck on submerged rocks and are often lost during plant sampling and preparation [49]. In spite of the different environments where the species live, the traps of all taxa are anatomically and functionally similar [21,76,77]. Heslop-Harrison [78,79] argued that the traps of terrestrial species are passive and only those of aquatic species, i.e., around 20% of the species [20,80], are active. Most studies of prey spectra were published since the 19th century [58,71], and focused on aquatic *Utricularia* (e.g., [81,82]), using mainly European species (e.g., [83,84,85]). Only a few studies were on terrestrial species [86,87], confirming their ability to catch prey. The diet commonly includes micro-crustaceans, nematodes, rotifers, and insect larvae [84]. Astonishingly, tadpoles [9] (Plate 20) and fish larvae [88,89], which are sometimes even bigger than the traps, were also reported as having been captured by *Utricularia* traps.

Darwin recognized some micro-organisms inside the traps that were not killed: “In all cases the bladders with decayed remains swarmed with living Algae of many kinds, Infusoria, and other low organisms, which evidently lived as intruders” [58] (p. 405). According to Hegner [90], who observed the protozoan fauna in *Utricularia* traps, the ciliates are not intruders but captives, and some can therefore be killed and possibly digested. Nevertheless, it is currently supposed that bacteria, algae, and protozoa live inside the traps as commensals, since the traps supply the bacteria with organic carbon, as commonly found in exudates of plant roots [91]. Furthermore, ciliates might feed on the debris and control the bacteria biomass inside the traps [83]. Recent reports outlined that bacteria, algae, protozoans, and rotifers live in anoxic conditions and in the presence of phosphatases [13,91,92,93]. Inside the traps, the concentration of nutrients suggests a complex dynamic between the exudates produced by the plant and trap’s community, whereas N, P, and C exudation is important for the development and support of the microbial community associated with the traps [92,94]. Moreover, it is known that the prey-free traps do not uptake N and P from the fluid inside the vesicles, but conversely exude an amount of N and P to the trap fluid to support their microbial communities [95]. In this respect, the trap micro-organisms behave more as parasites than simple commensals and represent an additional nutritional cost for the trap maintenance [92,96,97]. According to Sirová et al. [98], the traps act as microbial cultivators or farms, which aggregate complex microbial consortia, working to convert complex organic matter into a source of utilizable nutrients for the plants. Therefore, the micro-organisms inside the trap lumen are not to be considered simple “intruders,” as advocated by Darwin.

## 5. Perspectives and Future Directions

**Figure 2:**
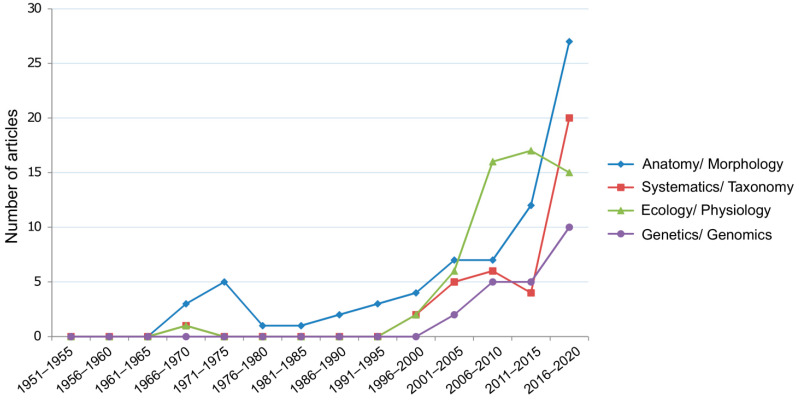
Number of scientific articles published between 1951 and 2020 referring to *Utricularia* and separated by research areas: anatomy and morphology, systematics and taxonomy, ecology and physiology, genetics and genomics. Each spot presents all articles published by each five years. The literature survey was conducted in the Web of Science (Clarivate Analytics) using the keywords: “(anatom * or morphology * or architecture * or structure *) and (utricularia or bladderwort *)” for the topic Anatomy/Morphology, “(systematic * or taxonom * or phylogeny * or cladistic *) and (utricularia or bladderwort *)” for Systematics/Taxonomy, “(ecology * or physiology * or ecophysiology *) and (utricularia or bladderwort *)” for Ecology/Physiology, and “(genetic * or genomic * or transcriptome * or proteome *) and (utricularia or bladderwort *)” for Genetics/Genomics. Following this non-exhaustive survey, all papers were manually inspected of their title, abstract, and keywords.

From a historical point of view, various fields have increasingly contributed to a better understanding of *Utricularia* (Figure 2), particularly after 1995, mainly with regard to anatomy, morphology, and systematics/taxonomy, but also concerning ecological and nutritional aspects of carnivory. New insights on genetic mechanisms were provided with respect to the genome size [99,100,101,102] and developmental programs of organs (e.g., [69,103]). Thus, more genetic studies may answer questions regarding the unusual (dynamic, “fuzzy”) morphology of vegetative organs which have been debated since the works of Goebel [39,40] and Kamieński [34] (Figure 1B,C). As a consequence, it will be crucial to integrate information from these different approaches and methods in order to develop a better understanding of the complex processes related to ontogenesis and plant architecture in *Utricularia*.

Despite of some studies on post-seminal development [9,44,104,105], our understanding of the *Utricularia* seedling and the recognition of its structures, such as cotyledons or pseudocotyledons, is still vague. Therefore, more studies of post-seminal development of *Utricularia* species of different life forms (aquatic, terrestrial, rheophytic, lithophytic, and epiphytic) are needed to better understand the different observed patterns of seedling development, vegetative structures, and possible adaptations, from an evolutionary and phylogenetic perspective.

In recent decades, some studies were ground-breaking in depicting the architecture and mechanisms of traps. Investigations on trap anatomy, with the aim to recognize and characterize key structures [21,106,107,108,109,110] and the mechanisms of trap firing, i.e., triggering active suction [6,77,111,112,113,114,115,116], were important advances and showed that no matter whether the species are aquatic or terrestrial, the traps are similar in structure and function. Nevertheless, most observations were performed on aquatic species, and studies on terrestrial/amphiphytic, lithophytic, epiphytic, and rheophytic species are still missing. Going forward, further phylogenetic and morphogenetic research might help to reveal the evolutionary trends of different *Utricularia* lineages and thus explain the differences of traps among the taxa (e.g., [109,117]).

Studies showing the minute sizes of chromosomes [118] and genomes of *Utricularia* and *Genlisea* and their evolutionary aspects [99,100] (Figure 2) demonstrated the clear need for further research in this group of plants. Even though the genes that are present in the *Utricularia* genomes are comparable to those of other higher plants, with the noncoding DNA and transposable elements being the main responsible for genome size discrepancies [99,102], it is crucial to determine whether some key genes, such as for the development of root structures [99] or morphological simplification [119], are present or absent. Accordingly, transcriptome studies could uncover the genes and gene families playing a role in root control and morphogenesis. For example, some genes known to be involved in root development and control are expressed in the *U. vulgaris* transcriptome, and some of them are also present in the *U. gibba* genome [99,120]. Accordingly, the lack of roots may not be due to a loss of genes involved in root development but could also be the result of gene functional specialization co-opting orthologous genes during the evolution of *U. gibba*, with the heterotopic transfer of the function of root genes to other organs [121]. Similarly, the *U. gibba* genome indicated the absence of homologs of the WOX5 gene [121,122], which is responsible for controlling stem fate in root apical meristem [123], but the expansion of genes in the WUS-like subfamily could be associated with the specialized bauplan and ramified branching patterns common to the species. Nonetheless, when genomes of terrestrial species of *Utricularia* were explored, putative domains of WOX genes were identified (Silva et al., in prep.). The development of genetically transformed *Utricularia*, as already proposed for *U. gibba* [124], could be an important tool as a system for the expression of key genes to morphogenesis as the species present a very reduced genome.

However, to overcome this gap, and to advance in these important issues related to morphology simplification and loss of roots in Lentibulariaceae, we need a more complete scenario comprising more genomes and transcriptomes for *Utricularia*, particularly with the sampling of different life forms, and the inclusion of *Genlisea* as the other member of the rootless clade in Lentibulariaceae.
